# Supplementary material for: Nutritional Status and Diet Style Affect Cognitive Function in Alcoholic Liver Disease
Source: Nutrients. 2021 Jan 9;13(1):185. doi: 10.3390/nu13010185 (PMC7826807; doi:10.3390/nu13010185)
Supplement: Supplementary file 1 [file nutrients-13-00185-s001.pdf]

Supplementary Table S1. Neuropsychological tests

Variable	BMI <22 (n=17)	BMI ≥22 (n=26)	P- value
**K-MMSE**			
K-MMSE, orientation to time	4.5 (0.7)	4.5 (0.8)	0.951
K-MMSE, orientation to place	4.9 (0.2)	5.0 (0.2)	0.898
K-MMSE, attention and calculation	3.7 (1.4)	3.8 (1.3)	0.784
K-MMSE, recall	2.0 (0.9)	2.1 (0.8)	0.464
K-MMSE, drawing	0.8 (0.4)	0.8 (0.4)	0.746
K-MMSE, total score	26.4 (2.9)	27.1 (2.4)	0.248
**Attention**			
RCFT copy scores	72.1 (25.9)	62.8 (33.6)	0.162
**Language**			
Calculation total score	9.7 (2.5)	10.2 (2.5)	0.417
**Memory**			
SVLT recall total score	18.2 (5.4)	18.0 (4.4)	0.884
SVLT delayed recall	6.4 (2.7)	6.1 (2.5)	0.575
SVLT recognition score	19.7 (4.1)	18.0 (4.6)	0.085
RCFT immediate recall	16.7 (9.5)	16.7 (8.4)	0.989
RCFT delayed recall	15.7 (9.1)	17.4 (7.3)	0.360
RCFT recognition score	18.8 (3.0)	17.6 (3.8)	0.127

K-MMSE**,** Korea-mini mental status examination; SVLT, Seoul-verbal learning test; RCFT, Ray-complex figure test;
